# *Ephedra alata* Seeds Confer Kidney Protection against Early Life Exposure to Acephate by Regulating Oxidative Insult and Activating Autophagy

**DOI:** 10.3390/life13122254

**Published:** 2023-11-25

**Authors:** Afoua Mufti, Anouar Feriani, María del Mar Contreras, Saber Nehdi, Najla Hfaeidh, Nizar Tlili, Abdel Halim Harrath

**Affiliations:** 1 Laboratory of Biotechnology and Biomonitoring of the Environment and Oasis Ecosystems, Faculty of Sciences of Gafsa, University of Gafsa, Gafsa 2112, Tunisia; muftiafoua@yahoo.com (A.M.); ferianianwer@yahoo.fr (A.F.); najhfaiedh@yahoo.fr (N.H.); 2 Department of Chemical, Environmental and Materials Engineering and Centre for Advanced Studies in Earth Sciences, Energy and Environment (CEACTEMA), Universidad de Jaén, Campus Las Lagunillas, 23071 Jaén, Spain; mcgamez@ujaen.es; 3 Department of Zoology, College of Science, King Saud University, Riyadh 11451, Saudi Arabia; nehdisabeur@gmail.com; 4 Institut Supérieur des Sciences et Technologies de l’Environnement Borj Cédria, Université de Carthage, Hammam chat 2050, Ben Arous, Tunis 1073, Tunisia; nizar.tlili@fst.rnu.tn

**Keywords:** *Ephedra alata*, acephate, nephrotoxicity, autophagy, oxidative stress, molecular docking

## Abstract

The aim of the current work was to examine for the first time the nephropreventive capacity of *Ephedra alata* seed extract (E) against maternal exposure to acephate in rat offspring. The in vivo results revealed that *E. alata* supplementation for 28 days (40 mg/kg b.w.) significantly attenuated the nephrotoxicity in adult offspring induced by acephate. In fact, it decreased the levels of creatinine and uric acid and increased the albumin content compared to the intoxicated group. The in utero studies showed that *E. alata* inhibited the renal oxidative stress generated by acephate exposure by reducing lipid peroxidation and enhancing antioxidant biomarker activities (GSH, CAT, and SOD). The inhibition of DNA fragmentation and the improvement of the ultrastructural changes highlighted the prophylactic effect of *E. alata* in renal tissue. Additionally, the immunofluorescence study showed the upregulation of *LC3* gene expression, suggesting the capacity of *E. alata* extract to stimulate autophagic processes as a protective mechanism. Molecular docking analysis indicated that hexadecasphinganine, the major compound in *E. alata*, has a higher affinity toward the Na^+^/K^+^-ATPase, epithelial sodium channel (ENaC), and sodium hydrogen exchanger 3 (NHE3) genes than acephate. Hexadecasphinganine could be considered a potential inhibitor of the activity of these genes and therefore exerted its preventive capacity. The obtained findings confirmed that *E. alata* seed extract exerted nephropreventive capacities, which could be related to its bioactive compounds, which possess antioxidant activities.

## 1. Introduction

The incidence of chronic kidney disease has been increasing at a worrying rate in recent decades. More than 10% of the world’s population suffers from at least one form of kidney disease [1]. Increased blood pressure, diabetes mellitus, and obesity are common risk factors [2]. However, nearly 40% of pathological cases have been associated with environmental risk factors such as heavy metals, nanomaterials, and mostly agrochemical exposure (pesticides and fertilizers) [3]. Several studies have shown that various classes of pesticides can develop the main risk factors that cause chronic kidney illnesses [4]. Acephate, as an organophosphate insecticide, is considered to be the most frequently used pesticide in agriculture. According to the WHO, food and drinking water are the main sources of exposure to this pesticide, which increases the risk of multiorgan anomalies [5,6]. Furthermore, the evidence reflects that prenatal exposure to pesticides affects renal function and causes chronic kidney diseases in adulthood [7,8]. A recent experimental study reported by [7] showed that in utero acephate exposure affects kidney function in adult rat offspring.

Currently, the drugs used to treat kidney disorders are sometimes insufficient and can have serious side effects [9]. In view of this observation, research is moving toward medicinal plants, considered an important source of multiple phytotherapeutic substances endowed with biological activities, and possibly considered as efficacy weapons, making it possible to counteract renal oxidative stress and its damage [10,11].

Among the medicinal species of Tunisia is *Ephedra alata*, an endemic desert species that belongs to the Ephedraceae family and is an herbaceous plant growing in rocky mountains. The leaves, stems and fruits of this plant are used in traditional medicine as active drugs against various ailments [12]. The scientific literature regarding the pharmacological characteristics of *E. alata* revealed varied beneficial properties of the extracts of this species, such as antioxidant, anticancer, and antiviral capacities [13,14]. In fact, the explored potential health benefits are related to their active molecules [13,15]. Recent studies by Alqahtani et al. [7] highlighted the phytochemical contents of *E. alata* fruits. HPLC–ESI–QTOF/MS analysis revealed the presence of various metabolites, including fatty acids, flavonoids, sphingolipids, and ephedrine derivatives (Table S1).

Despite the available knowledge about the phytochemical profile of *E. alata* seeds and their biological activity in vitro, the in vivo study of this plant remains unknown and needs to be explored. Thus, this is the first investigation to evaluate the preventive capacity of *E*. *alata* seed extract against the effect of prenatal intake of acephate on kidney function in adult rat offspring. A molecular docking analysis was used to show the interaction of hexadecasphinganine, the major compound in *E. alata*, with Na^+^/K^+^-ATPase, ENaC, and *NHE3* genes, as important biomarkers implicated in acephate-induced nephrotoxicity.

## 2. Materials and Methods

### 2.1. Samples: Extraction and Analysis

The extract of *Ephedra alata* seeds (E) was obtained using the methodology reported in a previous study [16]. Briefly, 1 g of dried *E. alata* seeds was extracted using 40 mL of methanol:water (80:20, *v*/*v*) in an ultrasonic bath for 60 min. The obtained extract was centrifuged (1000× *g* for 15 min) and then evaporated under reduced pressure at 40–50 °C to remove water and methanol. The final extract (E) was kept at −80 °C for the in vivo experimental test. In a recent study, E was characterized using HPLC-ESI-QTOF-MS/MS [7]. The obtained results are summarized in Table S1. Chromatographic analysis revealed the richness of *Ephedra* extract with bioactive components, including ephedrine derivatives, sphingolipides, flavonols, hexosides, and fatty acids (Table S1). The predominant bioactive compound was hexadecasphinganine (Figure S1).

### 2.2. Animal Model

Healthy mature Wistar rats (200–250 g of weight and 60 days old), purchased from a Central Pharmacy of Tunisia, were used in this study. Animals were kept in sets of polypropylene plastic cages under standard conditions (12 h blackness/day schedule) at 23 ± 1 °C. The rats had free access to water and ordinary laboratory food from the animal nutrition company “SNA”, Sfax, Tunisia). All investigations were determined according to the Ethical Committee for the Care and Use of Laboratory Animals at the University of Gafsa, Tunisia (No: FSG-AE-20-23).

### 2.3. Experimental Design

The rats were allowed one week to acclimatize, weighed, and randomly separated into five groups (six animals in each group, n = 6):

Group I (Control): the rats received gavage treatment with vehicle control (corn oil, Sigma Aldrich, St. Louis, MO, USA).

In group II (ACP-1) and group III (ACP-2), the animals were treated daily by gavage with acephate (Figure S2) (Sigma Aldrich, 98% purity, CAS Number: 30560-19-1) dissolved in corn oil at doses of 14 and 28 mg/kg body weight, respectively, from Day 6 of pregnancy, which corresponds to the implantation of the fetus, until delivery.

Group IV (E + ACP-1) and group V (E + ACP-2) rats were pretreated by gavage with E (40 mg/kg body weight) dissolved in corn oil for 28 days and received acephate (14 and 28 mg/kg b.w., respectively) daily from Day 6 of pregnancy.

All the groups treated with acephate were given 14 mg/kg and 28 mg/kg of body weight according to our recent report [7] and based on the oral LD50 values of 1/10 and 1/20 of acephate, respectively. To verify the absence of *E. alata* seed extract adverse effects in rats, four groups receiving multiple doses of E (5, 10, 20, and 40 mg/kg body weight) were compared to untreated rats within 24 h and during a subsequent 21 day observation period. No remarkable variation in the behavior or mortality of the animals was detected up to the selected dose of E (40 mg/kg body weight).

### 2.4. Biological Sample Collection

The pregnant rats were kept until normal delivery, and on postnatal week 8, the adult female rat offspring were euthanized. Blood was obtained from the orbital venous plexus. The plasma was separated by centrifugation (750× *g*; 10 min; 4 °C) and directly used for the analysis of biochemical parameters. Plasmatic creatinine (Cat# 80343. Range: 0.15–13.5 mg/dL; Crystal Chem Inc.; Elk Grove Village, IL, USA), uric acid (DIUA-250, range 0.22 mg/dL to 30 mg/dL; BioAssay Systems, Hayward, CA, USA), and albumin (ab235642; Abcam, Cambridge, MA, USA) were evaluated via assay kits following the manufacturer’s protocol. The animals were dissected, and then the kidneys were harvested, weighed, and washed with phosphate-buffered saline. Portions of these tissues were immediately fixed in neutral buffered formalin (NBF) for immunofluorescence and histopathological testing. The remaining tissue was directly fixed in RNAlater solution and then stored at −80 °C for oxidative stress analysis and molecular studies.

### 2.5. Analysis of DNA Fragmentation

To detect DNA damage, the protocol reported in a recent study was used [16]. Briefly, 60 mg of renal tissue was rinsed with cold PBS and then crushed in digestion buffer that contained proteinase K. After incubation at 56 °C for 3 h, the DNA was isolated by adding a volume of phenol/chloroform/isoamyl alcohol (25 V/24 V/1 V). The obtained DNA was resuspended in 5 µL of RNase (Sigma–Aldrich) to degrade all cellular RNAs. The isolated DNA was stained with ethidium bromide and then subjected to electrophoresis on an agarose gel (0.8%). The obtained gels were visualized under UV light and photographed.

### 2.6. Determination of Lipid Peroxidation

Approximately 0.5 g of excised renal tissues was homogenized into 2 mL ice-cold physiological saline (pH 7.4), sonicated twice, and centrifuged for 20 min at 4000× *g* (4 °C). The collected supernatants were used to determine the levels of malondialdehyde (MDA) using a thiobarbituric acid reaction at an absorbance of 532 nm in a spectrophotometer (Shimadzu, Kyoto, Japan, UV-1800). The results were expressed as nmol MDA/mg of protein, as described previously by Buege [17].

### 2.7. Evaluation of Enzymatic and Non Enzymatic Antioxidants

The GSH content in the kidney tissue was evaluated as reported by Aebi [18]. The activity of catalase (CAT) was calculated according to Sedlak and Lindsay [19], and the results are expressed as units of catalase activity (mg protein). The total activity of SOD was evaluated by measuring the inhibition of pyrogallol autoxidation catalyzed by superoxide radicals following the assay reported by Marklund and Marklund [20]. The protein levels of the renal tissue were measured [21] using bovine serum albumin as a reference.

### 2.8. Histopathological Examination

Kidney tissues were fixed in neutral buffered formalin (NBF) for 7 days and then introduced into paraffin liquid. The obtained blocks were cut into 4 μm sections and stained with hematoxylin and eosin (HE) to evaluate changes in renal structure. Processing and photomicrography were applied at the pathological anatomy laboratory at the Hospital, Gafsa, Tunisia, under a light microscope. All of the procedure was carried out according to our recent studies [7,22].

### 2.9. Immunofluorescence Examination

To explore the autophagy process of the renal tissue, an immunofluorescence study using a biomarker gene, namely, *LC3*, was performed as described in a previous study [23]. Briefly, the kidney tissues were sectioned at a thickness of 3 µm, dewaxed, hydrated, and washed with distilled water followed by PBS. Then, the slides were placed in a solution containing Triton X-100 and sodium citrate. The samples were blocked with FBS and incubated overnight at 4 °C with a primary antibody anti-LC-3 (1:100 dilutions, Dg-Peptide Co., Hangzhou, China), followed by subsequent incubation in the dark (45 min at 37 °C) with an anti-rabbit FITC-conjugated secondary antibody (1:2000 dilutions, Abcam, USA). Finally, the sections were mounted with Hoechst solution (diluted 1:15,000, Hoechst 33342, Life Technologies, Carlsbad, CA, USA) after treatment with TE buffer and PBS. The immunofluorescence sections were analyzed via a confocal microscope from Zeiss and then photographed. The mean fluorescence intensity for the protein expression of LC-3 was recorded by Zen service (ZEN lite) and quantified using GraphPad Prism 9 (GraphPad Prism 10.0.3 Software).

### 2.10. Molecular Docking Study

The three-dimensional crystallographic structures of the proteins were obtained from the RCSB Protein Data Bank (PDB) (https://www.rcsb.org/, accessed on 12 November 2023). Three proteins were selected for the docking process; they were selected based on their role in the induction of nephrotoxicity caused by acephate. They include the structure of the human epithelial sodium channel (ENaC) (PDB ID: 6BQN, resolution, 3.90 Å), the Na,K-ATPase in the E2P state with bound ouabain and Mg^2+^ in the cation-binding site (PDP ID: 4HYT, resolution 3.404 Å), and the crystal structure of a human NHE3-CHP1 (PDB ID: 7X2U, resolution, 3.20 Å).

Two compounds (ligands) were used for docking: the major compound of *E. alata* seed extract (hexadecasphinganine) (Figure S1) and the toxicant acephate (Figure S2). Compound 3D structures were obtained from the PubChem database (https://pubchem.ncbi.nlm.nih.gov/, accessed on 12 November 2023) under the following IDs:656816 and 1982.

Prior to the docking process, Schrodinger’s Protein Preparation Wizard tool [24] was used for 3D structures of proteins prepared by the addition of hydrogen atoms, the removal of water molecules and heteroatoms, the addition of required charges, the structure of protonation, and energy minimization via the OPLSe3 force field.

The essential part of the transmembrane region that harbors the pathway for ion conduction in ENaC was targeted in molecular docking [25,26]. The active pocket of Na, K-ATPase was determined according to a previous publication [27], while the active site of NHE3-CHP1 (PDB ID: 7X2U) was determined according to the position of the co-crystalized ligand.

Schrodinger’s Induced Fit Docking (in which protein residues are flexible) was used to study the possible interaction between protein active site residues and ligands. The ligands were kept flexible while the protein residues were kept flexible during the docking process, and 10 conformations were generated from each run. The XP docking score (XP Dscore), docking score (D Score) and energy model (emodel) were calculated to estimate the binding affinity of ligands in the protein active site. Extraprecision (XP) docking and scoring, a more potent and precise method, is intended to be applied to ligand poses with high scores. The Glide method of the Schrodinger suite uses the emodel score, one of the important molecular docking scoring systems, to select the most appropriate ligands. The GlideScore is a crucial way to distinguish between active and inactive small molecules with high binding capacity and others with weak or no binding affinities [28]. More negative scores indicate better stability of the complex.

### 2.11. Statistics

The distribution of data was assessed using Levene’s test. When the assumption of normality was met, a one-way analysis of variance (ANOVA) followed by Tukey’s test was performed with significance level set at <0.05. The results are expressed as the mean ± standard deviation (SD).

## 3. Results

### 3.1. Clinical Observation and Mortality

**Figure 1:**
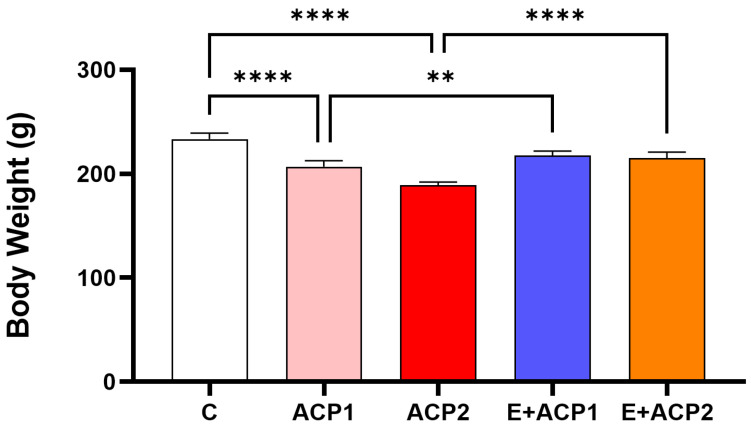
The effect of treatment on body weight on the different experimental groups of rat. The values are expressed as the mean ± standard deviation of six rats from each group. ** *p* < 0.01; **** *p* < 0.0001 significant differences. ACP1: low dose of acephate; ACP2: high dose of acephate; E + ACP1: *Ephedra* extract plus low dose acephate; E + ACP2: *Ephedra* extract plus higher dose of acephate.

There were no apparent clinical signs or deaths observed in any of the groups of animals at postnatal week 8. In fact, none of the animals showed any signs of toxicity in various aspects such as fur, eyes, sleep, skin, salivation, behavior, and diarrhea. The water and food consumption of the experimental rats did not differ significantly from that of the normal animals. However, there was a significant weight loss observed in the animals treated with ACP1 and ACP2 compared to the normal group, as depicted in Figure 1. The administration of *E. alata* extract partially attenuated the reduced body weight, as compared to the ACP1 and ACP2 group alone.

### 3.2. Assessment of Biochemical Renal Biomarkers

**Figure 2:**
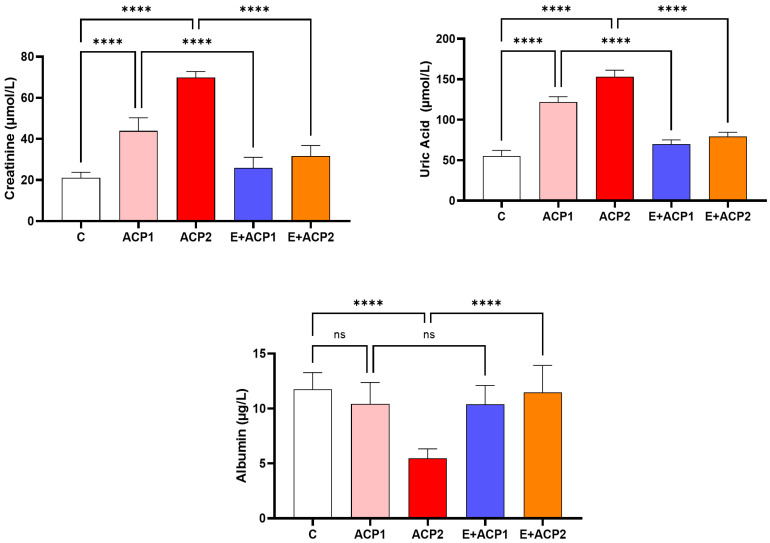
Variations in the concentrations of creatinine, uric acid, and albumin in the plasma of the offspring of female rats in the control and treated groups. The values are expressed as the mean ± standard deviation of six rats from each group. **** *p* < 0.0001 significant differences. n.s., not significant. ACP1: low dose of acephate; ACP2: high dose of acephate; E + ACP1: *Ephedra* extract plus low dose acephate; E + ACP2: *Ephedra* extract plus higher dose of acephate.

The variations in the biochemical biomarkers in plasma (Figure 2) revealed that the two doses of acephate (ACP1 and ACP2) caused a significant increase (*p* ≤ 0.001) in the levels of creatinine and uric acid, simultaneously with a remarkable decrease in albumin concentration in the offspring of female rats, in comparison to the normal group. However, in the (E + ACP1) or (E + ACP2) group, pretreatment with the *E. alata* seed extract (40 mg/kg) partially restored these plasmatic renal biomarkers without reaching those of the controls (C).

### 3.3. Evaluation of Lipid Peroxidation

**Figure 3:**
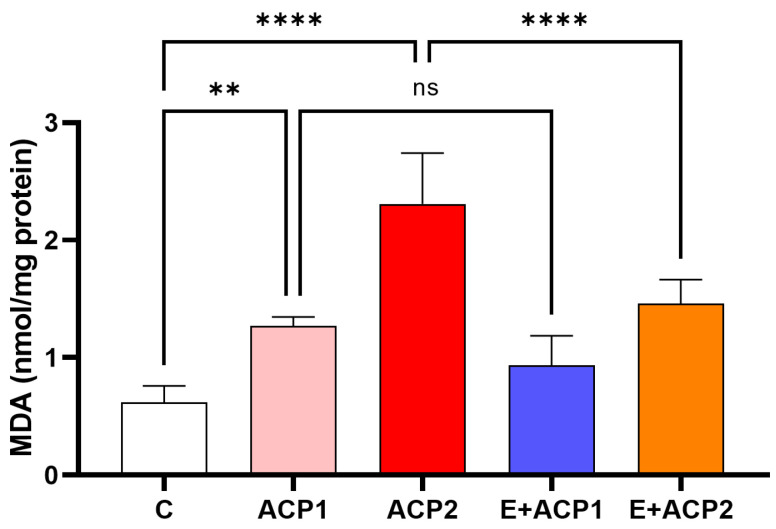
Variations in MDA content in the kidneys of control (C) and treated (ACP1, ACP2, E + ACP1 and E + ACP2) rats. The values are expressed as the mean ± standard deviation of six rats from each group. ** *p* < 0.01; **** *p* < 0.0001 significant differences. n.s., not significant. ACP1: low dose of acephate; ACP2: high dose of acephate; E + ACP1: *Ephedra* extract plus low dose acephate; E + ACP2: *Ephedra* extract plus higher dose of acephate.

The analysis of the results displayed in Figure 3 revealed a remarkable increase in the concentration of MDAn in the ACP1 (*p* < 0.01) and ACP2 (*p* < 0.0001) groups compared to the control group (C). However, the daily administration of *E. alata* clearly reduced lipid peroxidation in the renal tissue of the offspring of female rats through a decrease (*p* < 0.0001) in MDA levels by, ca., 40.2% in the E + ACP2 group compared to the ACP2 group. No significant effect was shown in the E + ACP1 group compared to the ACP1 group.

### 3.4. Variations in the Activity of Antioxidant Enzymes (SOD and CAT) and Total GSH Content

**Figure 4:**
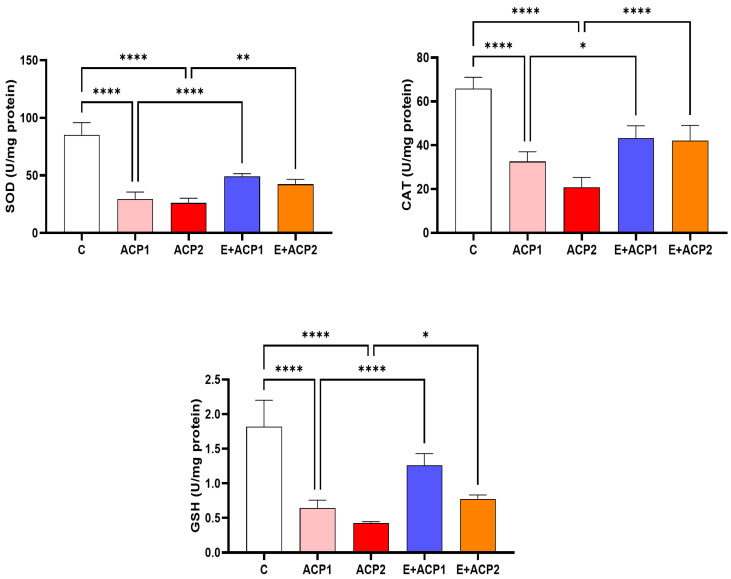
Changes in enzymatic (SOD and CAT) and nonenzymatic (GSH) antioxidants in renal tissue in the offspring of female rats in the control and treated groups. The values are expressed as the mean ± standard deviation of six rats from each group. * *p* < 0.05; ** *p* < 0.01; **** *p* < 0.0001 significant differences. n.s., not significant. ACP1: low dose of acephate; ACP2: high dose of acephate; E + ACP1: *Ephedra* extract plus low dose acephate; E + ACP2: *Ephedra* extract plus higher dose of acephate.

Figure 4 illustrates the activity of the antioxidant enzymes catalase (CAT) and superoxide dismutase (SOD) as well as the level of GSH in the kidneys of the different groups. 

A significant decrease in the activity of the targeted enzymes in the rats treated with both doses of acephate (ACP1 and ACP2) was observed. However, pretreatment with the extract of *E. alata* demonstrated a clear restoration of the activity of these enzymes in the renal supernatant. Likewise, the total GSH content in kidney tissue fell in the acephate-treated groups when compared to the control group. In contrast, a significant restoration of the level of this nonenzymatic antioxidant was observed in the E + ACP1 and E + ACP2 groups.

### 3.5. Genotoxicity Study

**Figure 5:**
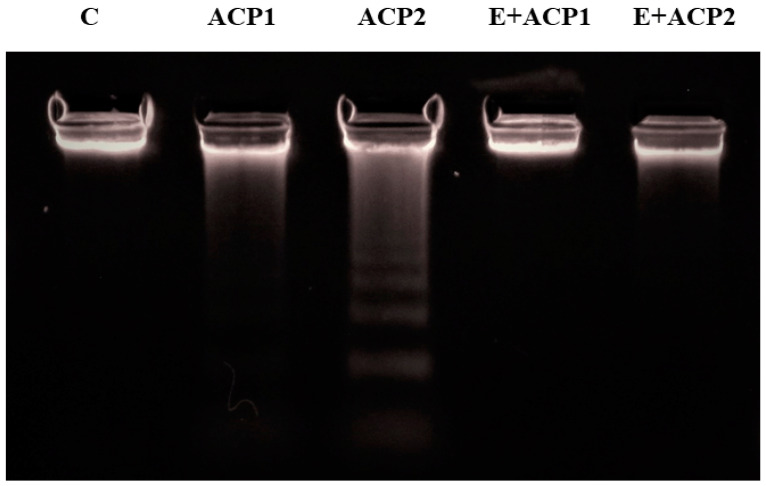
Agarose gel electrophoresis (2%) of DNA obtained from the kidney tissue of control rats (C), rats exposed to a low dose of acephate (ACP1), rats exposed to a high dose of acephate (ACP2), and rats pretreated with methanolic extract of *Ephedra alata* and exposed either to a low dose of acephate (E + ACP1) or a high dose of acephate (E + ACP2).

The results of genomic DNA analysis using agarose gel electrophoresis are summarized in Figure 5. The prenatal administration of acephate induced genotoxicity, resulting in DNA fragmentation of kidney tissue. Indeed, it was remarkable that following the administration of this insecticide, a smear appeared representing the DNA fragments at different molecular weights (ACP1 and ACP2) in comparison to the DNA isolated from the control group (C), which demonstrated a normal structure. However, pretreatment of the animals with the methanolic extract of *E. alata* at 40 mg/kg attenuated the effect of acephate-induced genotoxicity (E + ACP1 and E + ACP2).

### 3.6. Autophagy Analysis

**Figure 6:**
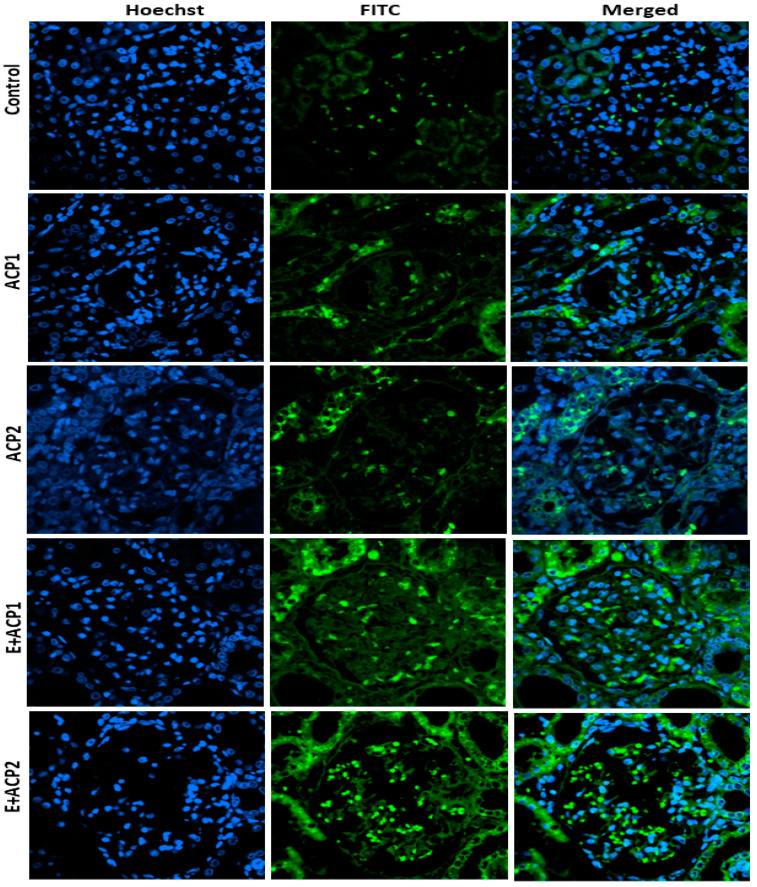

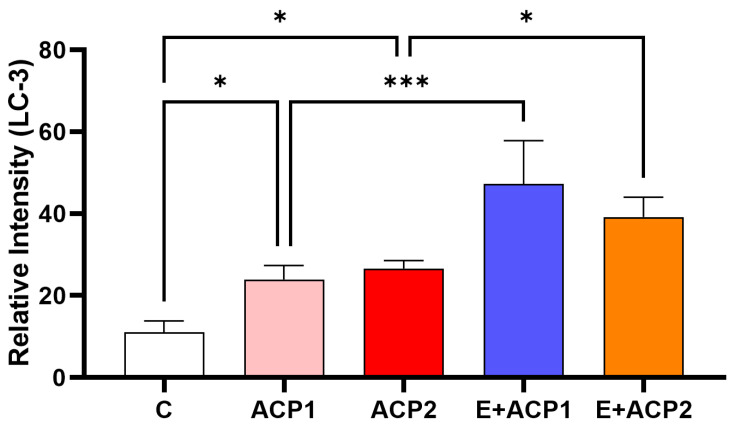
Quantification of the autophagic marker LC-3 in the offspring of control and treated rats. Immunofluorescence staining (Gx200), and relative intensity of LC-3. The values are expressed as the mean ± standard deviation of six rats from each group. * *p* < 0.05; *** *p* < 0.001 significant differences. ACP1: low dose of acephate; ACP2: high dose of acephate; E + ACP1: *Ephedra* extract plus low dose acephate; E + ACP2: *Ephedra extract* plus higher dose of acephate.

The microscopic observation of immunofluorescence sections, performed on the kidneys of control rats (Figure 6), showed a normal structure with minor traces of the *LC-3* gene. In the rats treated with the two doses of acephate, microscopic observation of the sections revealed a significant increase in the expression thresholds of *LC-3* in the kidneys relative to the control group. This was confirmed by the presence of green-colored granules in the two treated groups compared to the control group (Figure 6, ACP1 and ACP2). As a corollary, the treatment of rats with the extract of the seeds of *E. alata* (40 mg/kg) resulted in an improvement in green staining intensity (E + ACP1 and E + ACP2), thus reflecting an increase in protein expression (LC-3), which translated to an increase in autophagy, which acts, in this case, as a corrective mechanism.

### 3.7. Structural Exploration (H-E Staining)

**Figure 7:**
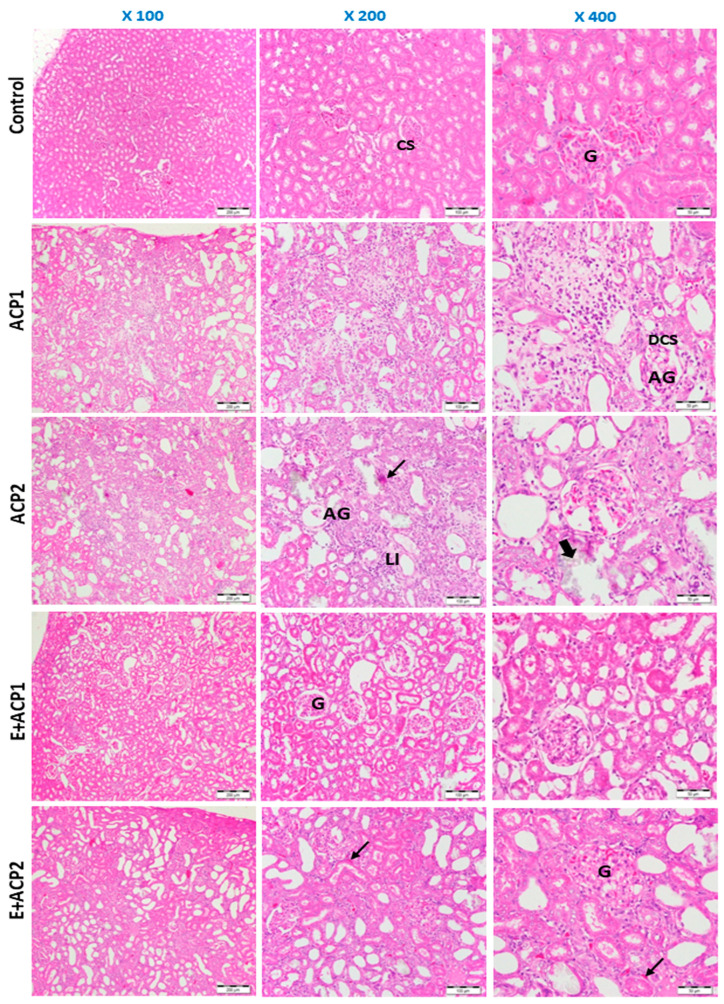
Photomicrographs of the kidney tissue of the experimental groups using hematoxylin and eosin staining (100×; 200×; 400×). ACP1: low dose of acephate; ACP2: high dose of acephate; E + ACP1: Ephedra plus low dose acephate; E + ACP2: Ephedra plus higher dose of acephate. Normal glomeruli (G), capsule space (CS), atrophy of glomeruli (AG), dilatation of capsule space (DCS), calcification (large arrow), renal cell fibrosis (arrow), excessive leukocyte infiltration (LI).

Figure 7 shows the histological sections of the kidney tissues stained with hematoxylin and eosin (H-E) in the different experimental groups. The results demonstrated that in control rats, the renal tissue presented the form of a renal cortex containing numerous glomeruli. Each glomerulus presented a bundle of looped capillaries surrounded by narrow Bowman’s spaces, with convoluted and intact distal and proximal tubules. However, sections of the kidneys in rats treated with both doses of acephate (ACP1 and ACP2) demonstrated marked changes reflecting the presence of a number of irregularly arranged glomeruli exhibiting fibrosis. Furthermore, areas of calcification, tubular dilation, and a high level of inflammatory cell infiltration in the tubular lumen and collecting duct were detected. However, it should be noted that the association of the methanolic extract of *E. alata* with acephate (E + ACP1 and/or E + ACP2) revealed protection against the toxicity of this product, as manifested by an amelioration of the structural damage. Indeed, an improvement in the architecture of the glomeruli was observed with the attenuation of the number of tubular dilatations, the regression of the areas of fibrosis, and the restoration of the number of inflammatory cells.

### 3.8. Molecular Docking Analysis

**Figure 8:**
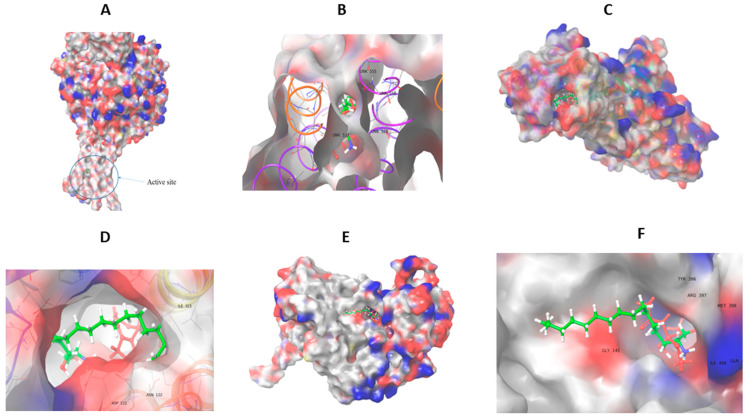
3D structure representation of the competitive interactions of ENaC (**A**,**B**), Na, K-ATPase (**C**,**D**), NHE3 (**E**,**F**), and the ligands hexadecasphinganine (green and white) and acephate (red). The blue color indicates basic residues; the red color indicates acidic residues. The active site is shown in a circle.

The robust technique of molecular docking is used to examine the atomic-level interactions of small compounds with proteins. In the current work, molecular docking was used to study the possible competitive interaction of hexadecasphinganine and acephate in the active site of the ENaC protein. The interaction of hexadecasphinganine and ENaC generated −6.05 kcal/mol docking energy, −41.05 kcal/mol glide emodel, and Glide van der Waals energies (EvdW) was −30.6; the sum of these energies resulted in this high affinity of ligand to protein residues. Additionally, the terminal polar part of the hexadecasphinganine which contains nitrogen and oxygen atoms donated three hydrogen bonds to oxygen in residues number 555 (two hydron bonds) and 59. While the middle nonpolar part of the ligand generated eight hydrophobic bonds with residues in hydrophobic protein pockets (552 (two), 66 (two), 528 (three), and 63); this results in tighter binding. The interaction of acephate and ENaC generated −3.5 kcal/mol, −35 kcal/mol, and evdw −33.6 Kcal/mol. When compared to hexadecasphinganine, the acephate formed only three hydrophobic bonds that were observed with residues number 555, 528, and 63 (Table 1) (Figure 8A,B).

The competitive interaction of Na, K-ATPase with both hexadecasphinganine and acephate exhibited docking energies of −5 and −3.08 kcal/mol, respectively, and emodel energies of −48.3 and −29.9 kcal/mol, respectively (Table 1). The polar part of the hexadecasphinganine donated four hydrogen atoms to oxygen atoms of residues Glu117 (two bonds), Asp121, and Thr797 in the hydrophilic part of Na, K-ATPase. Also, this part generated two salt bridges with the nitrogen atom of the ligand and oxygen atoms of (Glu117, and Asp121) of the protein; moreover one hydrophobic bond was observed with Lys905. The acephate and Na, K-ATPase generated two hydrogen bonds with Asn122 (Table 2), the small number of bonds and the lowest values of binding energy reflect the weaker interactions of protein and acephate.

Seven hydrophobic bonds were formed due to the interaction of Gln111, Glu116, Leu311, Glu312, Ile315 (two), and Leu793 due to the interaction of Na, K-ATPase and hexadecasphinganine. The interaction of Na, K-ATPase and acephate generated no hydrophobic bonds (Table 3) (Figure 8C,D).

The competitive interaction of hexadecasphinganine and acephate with NHE3 protein generated XP docking scores of −4.3 and −2.7 kcal/mol, and the emodel energies were −40.5 and −30.5, respectively (Table 1). The interaction of NHE3 and hexadecasphinganine exhibited six hydrogen bonds with Arg397 (two bonds), Met398, Gln497, and Ile498 (two bonds) (Table 2). NHE3 and hexadecasphinganine further generated four hydrophobic bonds with Leu144, Leu145, and Tyr396 (two bonds). NHE3 and acephate generated three hydrogen bonds with Thr142, Tyr396, and Arg397. Additionally, four acephate hydrophobic bonds were generated with Leu393, Tyr396, and Arg397 (Table 3) (Figure 8E,F).

## 4. Discussion

Currently, bio-antioxidants are attracting research interest due to their safety and beneficial effects against many diseases linked to oxidative stress [29]. Many medicinal plants contain large quantities of antioxidants capable of attenuating and neutralizing free radicals [16,30]. *E. alata* is one of the most commonly used plants in North African regions for the treatment of diabetes, cardiovascular diseases, and various cancerous lesions. However, no data are available on the nephroprotective effects of this species against pesticide toxicity. Therefore, this study explored for the first time the ameliorative effect of the methanol extract of *E. alata* seeds against the damage caused by in utero acephate exposure in the kidney tissue of the offspring of Wistar rats.

The administration of acephate caused renal dysfunction, marked by an increase in renal biomarkers, namely, creatinine, uric acid, and urea. These observed abnormalities have also been reported in rats exposed to other organophosphate compounds [31,32]. The significant increase in plasma levels of creatinine, urea, and uric acid observed could be associated with impaired glomerular function and renal tubular damage, which was confirmed by the histopathological results obtained in this study. The renal section demonstrated increasing numbers of sclerotic glomeruli, fibrosis, severe tubular dilations, and leukocyte infiltration in the offspring of rats treated with acephate.

The pre-co-administration of *E. alata* extract restored plasma concentrations of urea, uric acid, and creatinine to normal limits. The preventive capacity of *E. alata* seed extract was probably the result of improved renal membrane structure and subsequently the proper functioning of this tissue. The protective effect of *E. alata* could be linked to its content of phytochemical compounds. Indeed, recent reports have addressed the protective effect of natural compounds against kidney damage [33,34].

The generation of reactive oxygen species (ROS) (superoxide anion, hydroxyl radical, hydrogen peroxide, nitric oxide) is part of the normal metabolism of the cell. During contamination by a xenobiotic, an imbalance is created between the generation of ROS and the production of antioxidants, leading to a state of oxidative stress. Recent studies have revealed that the induction of oxidative stress and the disruption of total antioxidant capacity are possible mechanisms of toxicity of different pesticides in animals [35,36]. The present data revealed an increase in the level of lipid peroxidation (high MDA level in groups treated with acephate), consequently confirming the appearance of oxidative damage in the kidney tissues of offspring rats. Indeed, acephate is a highly lipophilic compound that is capable of easily infiltrating the cell membrane and making membrane lipids and proteins vulnerable to oxidative damage [37]. The administration of *E. alata* improved the status of lipid peroxidation, clearly indicating its powerful chemopreventive and antioxidant capacity against renal oxidative stress induced by acephate in the offspring of adult rats. As concluded above, *E. alata* exhibited an antioxidant role through the presence of biomolecules known for their ROS scavenging capacities [7].

Furthermore, pre-co-administration with *E. alata* attenuated renal oxidative stress by increasing the activities of SOD and CAT, as well as the levels of GSH in the kidney. Thus, *E. alata* extract consumption in early life might develop cellular antioxidative defense, which leads to protection against renal structural injury induced by acephate exposure in utero in female offspring during adulthood. Similar results published by [38,39] demonstrated that *E. alata* presented a protective effect against oxidative stress in various tissues.

These findings suggested the important antioxidant and ROS scavenging effects of *E. alata* seeds, which effectively shielded the renal tissue from radical-induced depletion during acephate toxicity. The antioxidant properties of *E. alata* might be due to its richness in bioactive compounds. In fact, recent studies by [7] revealed that the HPLC- QTOF-MS/MS analysis of *E. alata* seeds showed various phytochemicals, including flavonoids, sphingolipids, and ephedrine derivatives. Natural antioxidants are capable of reducing free radical injuries by acting directly as a free radical scavenger and/or indirectly by activating enzymatic (SOD, CAT and GSH-Px) and nonenzymatic (GSH) antioxidant systems, which reduce lipid peroxidation in animals [40,41,42,43]. In addition, these natural compounds play an effective function in preserving mitochondrial homeostasis and therefore protecting against oxidative damage [44].

DNA fragmentation is one of the first events in apoptosis. In the present study, acephate was found to be capable of inducing DNA damage. This compound increased DNA fragmentation and the formation of intense bands (ladder) marking apoptotic damage in kidney cells. Data from published studies have linked pesticide administration to increased DNA fragmentation and therefore genotoxicity in the kidney [45,46]. Conversely, *E. alata* treatment at a dose of 40 mg/kg significantly attenuated the observed genotoxicity and decreased the alteration of the DNA profile induced by acephate exposure. Several mechanisms could explain this effect, some of which were linked to the presence of a high level of beneficial metabolites known for their inhibitor properties of DNA fragmentation in renal tissue, as reported by previous studies [47,48].

Currently, autophagy is considered a target of agrochemical pollutants in various tissues [49]. Specific apoptotic markers (Beclin 1 and LC3) were explored to verify the degree of autophagy processes. In the current work, the expression of LC-3 protein was increased in the kidneys of rat offspring. Similar findings were reported by other researchers [50,51], who revealed that several insecticides are able to induce autophagy. Interestingly, *E. alata* seed extract treatment markedly enhanced LC-3 biomarker expression in renal tissue compared to the acephate-treated group, suggesting that *E. alata* exhibited an ameliorative effect on the autophagy pathway as a protective mechanism against the stress state. Indeed, experimental studies revealed that diverse phytochemicals could activate the autophagy process, thereby preventing and treating chronic kidney disease [52,53]. The route by which the *E. alata* extract increased the LC-3 and autophagy processes remains to be determined.

Recent studies by our team showed that prenatal exposure to acephate promotes kidney function failure and induces nephrotoxicity in rat offspring via upregulation of the mRNA expression levels of the ENaC, NHE3, and Na^+^/K^+^-ATPase genes [7,22]. Herein, the objective was to estimate the binding affinity and the competitive inhibitory effect of hexadecasphinganine, as the major compound in *E. alata* seeds, and acephate to different targets, such as ENaC, Na^+^/K^+^-ATPase, and NHE3. The molecular docking study revealed the ability of hexadecasphinganine to block the interaction site of the pesticide (acephate). Indeed, hexadecasphinganine exhibited better docking affinity (−6.05, −5.00, and −4.3 kcal/mol to ENaC, Na^+^/K^+^-ATPase, and NHE3, respectively) than acephate (−3.5, −3.08, and −3.3 kcal/mol to ENaC, Na^+^/K^+^-ATPase, and NHE3, respectively). Additionally, these results were supported by the low negative (better) results of the emodel scoring, which is one of the salient molecular docking scoring functions. Hexadecasphinganine generated better emodel scores with ENaC, Na^+^/K^+^-ATPase, and NHE3 than acephate. The interaction of NHE3 and hexadecasphinganine exhibited two hydrogen bonds with Arg397. NHE3 and acephate generated only one hydrogen bond with Arg397, which indicated the possibility of competitive inhibition of hexadecasphinganine when compared to a weakly interacting acephate. These findings suggested that hexadecasphinganine might be able to block the interaction site of acephate with the targets and potentially reduce their toxicity.

## 5. Conclusions

This study provides compelling evidence that *E. alata* seed extract exhibits nephroprotective properties, particularly against early life exposure to the pesticide acephate. The protective mechanism appears to be primarily through the reduction of oxidative stress, enhancement of antioxidant activities, inhibition of DNA fragmentation, and stimulation of autophagic processes within renal tissue. Moreover, the molecular docking analysis suggested that hexadecasphinganine, a major compound found in *E. alata*, may act as a potential inhibitor of certain genes implicated in acephate-induced nephrotoxicity. This could be one of the primary ways the extract exerts its preventive effects. These findings may lead to further investigation into the potential use of *E. alata* and its bioactive compounds in the prevention and treatment of nephrotoxicity and possibly other pesticide-induced toxicities. They also emphasize the need for more advanced research into the therapeutic potential of other medicinal plants in mitigating the health impacts of environmental pollutants.

## Figures and Tables

**Table 1:** Docking scores of the interaction of ligands and proteins (Kcal/mol).

		ENaC	Na, K-ATPase	NHE3
Hexadecasphinganine	XP Dscore	−6.05	−5.00	−4.3
	Glide emodel	−41.05	−48.3	−40.5
	D Score	−6.01	−4.96	−2.7
	Glide evdw	−30.6	−21.0	−25.2
Acephate	XP Dscore	−3.5	−3.08	−3.3
	Glide emodel	−35	−29.9	−30.5
	D Score	−3.45	−3.08	−3.3
	Glide evdw	−33.6	−27.0	−26.9

**Table 2:** The generated Hydrogen bonds.

	Index	Residue	AA	Distance H-A	Distance D-A	Donor Angle	Donor Atom	Acceptor Atom
Na, K-ATPase _ Hexadecasphinganine	1	117A	GLU	1.98	2.92	165.26	15,524 [O3]	1520 [O3]
	2	117A	GLU	1.85	2.86	176.25	15,526 [N3]	1520 [O3]
	3	121A	ASP	1.68	2.65	164.9	15,525 [O3]	1580 [O-]
	4	797A	THR	2.42	3.34	150.65	11,886 [Nam]	15,525 [O3]
Na, K-ATPase _ Acephate	1	122A	ASN	1.9	2.89	165.87	15,529 [Nam]	1591 [O2]
	2	122A	ASN	1.85	2.82	159.03	1592 [Nam]	15,526 [O3]
NHE3_ Hexadecasphinganine	1	397A	ARG	3.38	3.71	101.24	5613 [Ng+]	11,726 [O3]
	2	397A	ARG	3.43	3.93	113.39	5614 [Ng+]	11,726 [O3]
	3	398A	MET	2.6	3.52	152.03	5628 [Nam]	11,727 [O3]
	4	497A	GLN	1.91	2.83	158.05	11,727 [O3]	7105 [O2]
	5	498A	ILE	2.06	3	165.58	11,726 [O3]	7122 [O2]
	6	498A	ILE	2.06	3.05	164.29	11,728 [N3]	7122 [O2]
NHE3_ Acephate	1	142A	THR	2.7	3.1	105.92	1614 [O3]	11,730 [O2]
	2	396A	TYR	2.04	3.01	159.35	11,731 [Nam]	5586 [O2]
	3	397A	ARG	3.33	3.81	111.1	5613 [Ng+]	11,729 [O2]

**Table 3:** The generated hydrophobic bonds.

	Index	Residue	AA	Distance	Ligand Atom	Protein Atom
Na, K-ATPase _ Hexadecasphinganine	1	111A	GLN	3.64	15,541	1437
	2	116A	GLU	3.98	15,542	1503
	3	311A	LEU	3.63	15,541	4475
	4	312A	GLU	3.91	15,536	4492
	5	315A	ILE	3.93	15,536	4534
	6	315A	ILE	3.8	15,539	4533
	7	793A	LEU	3.84	15,532	11834
Na, K-ATPase _ Acephate		-	-	-	-	-
NHE3_ Hexadecasphinganine	1	144A	LEU	3.62	11,744	1646
	2	145A	LEU	3.34	11,740	1667
	3	396A	TYR	3.78	11,729	5587
	4	396A	TYR	3.41	11,733	5590
NHE3_ Acephate	1	393A	LEU	3.77	11,733	5533
	2	396A	TYR	3.71	11,733	5587
	3	397A	ARG	3.93	11,733	5609

## Data Availability

The data that support the findings of this study are available from the corresponding author (Abdel Halim Harrath) upon reasonable request.

## Supplementary Materials

The following supporting information can be downloaded at: https://www.mdpi.com/article/10.3390/life13122254/s1, Figure S1: Chemical structure of Hexadecasphinganine. Figure S2: Chemical structure of acephate. Table S1. Compunds identified in *E. alata* seeds extract. Ref. [54] is cited in Supplementary Materials.
