# Supplementary material for: Ephedra alata Seeds Confer Kidney Protection against Early Life Exposure to Acephate by Regulating Oxidative Insult and Activating Autophagy
Source: Life (Basel). 2023 Nov 25;13(12):2254. doi: 10.3390/life13122254 (PMC10745092; doi:10.3390/life13122254)
Supplement: Supplementary file 1 [file life-13-02254-s001.zip › life-2661912-supplementary.pdf]

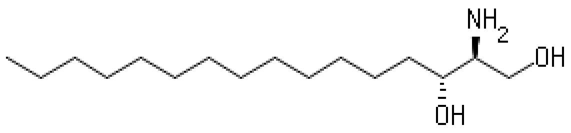

Figure S1: Chemical structure of Hexadecasphinganine

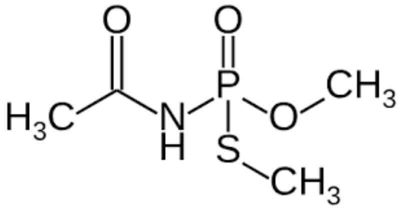

Figure S2: Chemical structure of acephate

Table S1.Compunds identified in *E. alata* seeds extract

No	Compounds [*]	Relative % [**]
**1**	Unknown	**6,48±0,13**
**2**	Methanoproline	1,25±0,09
**3**	Leucine/Isoleucine hexoside	1,04±0,05
**4**	Phenylalanine hexoside	1,09±0,16
**5**	Ephedrine derivative 1 (+ hexosyl + deoxyhexosyl)	2,16±0,08
**6**	Indoleacrylic acid	1,77±0,19
**7**	Hydroxykynurenic acid	2,09±0,38
**8**	Unknown	1,19±0,05
**9**	Kynurenic acid	1,45±0,11
**10**	Ephedrine derivative 2	0,33±0,00
**11**	Unknown	0,29±0,04
**12**	Ephedrine derivative 3	1,49±0,07
**13**	Isoschaftoside	0,43±0,01
**14**	Ephedrine derivative 4	2,84±0,16
**15**	Unknown	0,48±0,08
**16**	Unknown	0,92±0,11
**17**	Isorhamnetin *O*-hexoside-*O*-deoxyhexoside 1	0,19±0,02
**18**	Isorhamnetin *O*-hexoside-*O*-deoxyhexoside 2	0,23±0,02
**19**	Unknown (compound 18 + C6H11NO)	2,89±0,38
**20**	Kaempferol 3-*O*-rhamnoside	0,09±0,02
**21**	Unknown (compound 19 + C6H11NO)	1,38±0,20
**22**	Unknown (compound 20 + C6H11NO)	0,27±0,03
**23**	Unknown (compound 21+C6H11NO	0,03±0,00
**24**	Tetradecasphinganine	0,41±0,01
25	**Hexadecasphinganine**	**30,59±0,19**
**26**	Phytosphingosine	3,52±0,20
**27**	Sphingolipid derivative 1	4,36±0,63
**28**	Sphingolipid derivative 2	0,42±0,01
**29**	Unknown	0,44±0,03
**30**	Sphingolipid derivative 3	1,17±0,17

**31**	Unknown	3,10±0,14
**32**	9,10-Dihydroxystearic acid	0,72±0,07
**33**	Deoxysphinganine	0,46±0,04
**34**	Hydroxyhexadecanoic acid	0,34±0,02
**35**	Hydroxyoctadecatrienoic acid	0,54±0,01
**36**	Unknown (choline derivative)	3,64±0,07
**37**	Unknown	3,31±0,05
**38**	Unknown	**7,66±0,59**
**39**	Oleamide	**5,77±0,98**
**40**	N-Palmitoylsphingosine	0,33±0,00
**41**	Unknown	**2,84±0,16**

* [54]

** Results of the present study
